# Clusters of specialized detector cells provide sensitive and high fidelity receptor signaling in the intact endothelium

**DOI:** 10.1096/fj.201500090

**Published:** 2016-02-12

**Authors:** Calum Wilson, Christopher D. Saunter, John M. Girkin, John G. McCarron

**Affiliations:** * Strathclyde Institute of Pharmacy and Biomedical Sciences, University of Strathclyde, Glasgow, United Kingdom; and; † Centre for Advanced Instrumentation, Biophysical Sciences Institute, Department of Physics, Durham University, Durham, United Kingdom

**Keywords:** endothelial calcium signalling, microendoscopy, gradient index, calcium imaging, endothelial heterogeneity

## Abstract

Agonist-mediated signaling by the endothelium controls virtually all vascular functions. Because of the large diversity of agonists, each with varying concentrations, background noise often obscures individual cellular signals. How the endothelium distinguishes low-level fluctuations from noise and decodes and integrates physiologically relevant information remains unclear. Here, we recorded changes in intracellular Ca^2+^ concentrations in response to acetylcholine in areas encompassing hundreds of endothelial cells from inside intact pressurized arteries. Individual cells responded to acetylcholine with a concentration-dependent increase in Ca^2+^ signals spanning a single order of magnitude. Interestingly, however, intercellular response variation extended over 3 orders of magnitude of agonist concentration, thus crucially enhancing the collective bandwidth of endothelial responses to agonists. We also show the accuracy of this collective mode of detection is facilitated by spatially restricted clusters of comparably sensitive cells arising from heterogeneous receptor expression. Simultaneous stimulation of clusters triggered Ca^2+^ signals that were transmitted to neighboring cells in a manner that scaled with agonist concentration. Thus, the endothelium detects agonists by acting as a distributed sensing system. Specialized clusters of detector cells, analogous to relay nodes in modern communication networks, integrate populationwide inputs, and enable robust noise filtering for efficient high-fidelity signaling.—Wilson, C., Saunter, C. D., Girkin, J. M., McCarron, J. G. Clusters of specialized detector cells provide sensitive and high fidelity receptor signaling in the intact endothelium.

The vascular endothelium is a complex sensory system that responds to a large number of signaling molecules (activators) that arrive *via* blood, neurotransmission, smooth muscle, and from endothelial cells themselves to control vascular function. In this noisy chemical environment, concentrations of each activator change almost continuously, and the endothelium detects the alterations and evokes a vascular response. The detection and signaling systems involved are robust to random fluctuations (noise) that obscure the signals, and yet the cells are sensitive and able to discriminate very small changes in agonist concentration (1). The endothelium is also capable of responding to high concentrations of agonists. Thus even though sensitivity is high, the endothelium operates efficiently over a large concentration range and does not readily saturate. When each new concentration change has stabilized, the endothelium must detect signals from random fluctuations around the altered basal level. How, in the presence of substantial noise, the endothelium manages to sense fluctuations of activators just above basal levels while maintaining a graded response capable of detecting low and high concentrations is not known.

Agonist stimuli are transduced to changes in the endothelial Ca^2+^ concentration to coordinate the endothelium’s control of vascular tone. Ca^2+^ acts as a highly localized subcellular messenger and a multicellular communicator with wide reach (2–6) to communicate signals over distance. Cellular heterogeneity in Ca^2+^ responses is an important feature of the endothelium and may govern the nature of the tissue-level response to activation (1, 7–9). The precise physiologic significance of the heterogeneity is not fully understood. The physiologic configuration of arteries is also important in the endothelium’s responsiveness and sensitivity to agonists. For example, the sensitivity to vasoconstrictors decreases, and an important endothelial-derived hyperpolarizing response is absent in arteries stretched on wire myographs when compared with those held in a normal configuration and physiologic pressures (10–12).

Endothelial function in larger arteries, such as the carotid artery, is critical to normal function of the vasculature and to the development of cardiovascular disease (*e.g.,* atherosclerosis). The endothelium regulates the contractile response of the carotid artery (13–18) and exerts profound physiologic control of artery structure by controlling the proliferative status of the cells within the wall (19). Changes in the endothelium’s control of cell proliferation in the artery wall, as a result of agonist activation, result in arterial remodeling, intimal-medial thickening, and plaque formation in vascular disease (19). However, in larger arteries visualizing Ca^2+^ signaling in the endothelium in a physiologic configuration has been particularly challenging because of light scattering and substantial curvature of the artery wall.

To address how the endothelium detects agonist and coordinates Ca^2+^ signals across cells, to control artery function, we used a miniature fluorescence endoscope that was developed around a gradient index (GRIN) lens. The miniature fluorescence endoscope permitted Ca^2+^ signaling to be measured from inside the lumen of intact pressurized arteries while the vessel is in a physiologic configuration and at normal intraluminal pressure. The endoscope allows ∼200 endothelial cells to be imaged with subcellular resolution and has a high depth of field (141 µm) so that focus is maintained across the curved endothelial layer of the pressurized artery.

We show that agonist sensing is carried out by cells with various fixed concentration sensitivities that operate over a narrow concentration range. By merging multiple standard concentration responses, from various cells working over various predefined concentration dependencies, a sensitivity range for agonist sensing that is effective over at least 3 orders of magnitude of concentration is achieved. Cells of comparable sensitivity cluster (multicellular macrodomains) to provide agonist concentration sensory spaces. Simultaneously activating several cells in the cluster generate Ca^2+^ waves that are transmitted distances scaling with stimulus intensity. The clusters provide a coincidence detection system for noise rejection. The results show that the endothelium acts as a distributed sensing system with the response of each cell and cluster part of a collective decision on concentration; heterogenous cell sensitivity, clustering, and signal propagation provide an integrative mechanism for noise filtering and sensing agonists over wide concentrations ranges.

## MATERIALS AND METHODS

### Endothelial Ca^2+^ imaging in pressurized arteries

**Figure 1.**
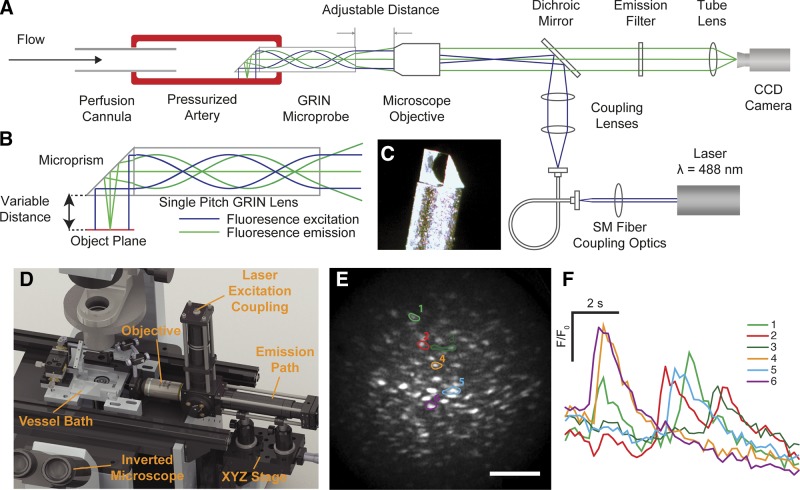
Intraluminal endothelial imaging. *A*) Simplified schematic diagram of the endothelial microendoscopy utilizing GRIN. *B*) Schematic illustrating the optical excitation and emission paths through the side-viewing GRIN microprobe. The GRIN reconjugates the image plane of a conventional microscope through the length of the cylinder (green lines). The collimated input excitation light is recollimated at the output of the GRIN (blue lines). *C*) An image of the distal end of the GRIN microprobe with the microprism attached. The GRIN is protected inside a stainless steel sheath. *D*) A 3-dimensional rendering of the system on an inverted microscope showing the custom vessel bath, position of the GRIN microprobe assembly, the objective lens, XYZ translation stage for positioning the artery, and excitation coupling and emission pathway. The laser and camera are outside the image. *E*) Fluorescence image of the endothelium visualized using the GRIN imaging system. Scale bar, 100 μm. *F*) Individual baseline corrected Ca^2+^ signals of corresponding ROIs (*E)* showing spontaneous Ca^2+^ transients.

Carotid arteries were obtained from male Sprague-Dawley rats [10–12 wk old; 250–350 gm; killed by overdose of pentobarbital sodium; Schedule 1 procedure; Animal (Scientific Procedures) Act 1986, United Kingdom]. The arteries were mounted onto stainless steel (22 gauge) cannulae in a custom vessel bath, and the endothelium was selectively loaded with a fluorescent Ca^2+^ indicator by perfusing the lumen with a Ca^2+^ indicator loading solution for 30 min at 37°C. After loading, arteries were removed from one of the cannulae and remounted on a custom microendoscope fluorescence imaging system to visualize the endothelium and maintain the arteries at physiologic pressures (**Fig. 1**).

We constructed the microendoscope imaging system around a side-viewing microprobe. The microprobe comprises a 30.2 mm long, 0.5 mm diameter GRIN (SRL-050; Nippon Sheet Glass, Tokyo, Japan) encased in a protective stainless steel sheath (0.7 mm outer diameter; Fig. 1). A 0.5 mm microprism (66-771; Edmund Optics, Barrington, NJ, USA) was fixed to one end of the GRIN to provide the side-viewing capability (Fig. 1). In contrast to other GRIN microendoscopes, used for surface imaging of heart and brain cells (20–24), a low numerical aperture (∼0.1) single-pitch GRIN with a correspondingly large depth of field (141 µm) was utilized to enable the entirety of the cross-sectional area of the GRIN to be used for imaging the curved inner (endothelial) surface of pressurized arteries [see Wilson *et. al.* (25) for a full description].

Fluorescence excitation, provided by a diode-pumped solid-state laser (488 nm), was coupled to the GRIN using a standard microscope objective (×20, 0.75 numerical aperture; Nikon, Tokyo, Japan) (Fig. 1). The laser light was focused at the back focal plane of the microscope objective such that the beam emerged from the microscope objective collimated to 0.5 mm and was then relayed onto the endothelium (0.5 mm diameter, 200 µW) by the side-viewing GRIN. This ensured the illumination area remained constant under probe refocusing (26). Fluorescence emission returning through the GRIN microprobe was collected by the same microscope objective and focused onto an sCMOS camera (Zyla 3-Tap, Belfast, United Kingdom). Images were acquired at 5 Hz using µManager software (*https://micro-manager.org/*) (27). The magnification of the microendoscope system was ×6.5, providing an effective pixel size of 1 µm at the object plane and permitting subcellular endothelial imaging (∼4.5 μm resolution) of a large number (∼200) of endothelial cells across the full 0.5 mm diameter of the GRIN (Fig. 1).

After loading and mounting, to confirm endothelial viability, endothelial Ca^2+^ signaling was induced by 100 µM extraluminally applied acetylcholine (ACh). The concentration of ACh at the lumen of the intact pressurized artery is likely to be ∼1000-fold less than the extraluminal bath ACh concentration (Supplemental Fig. 2). In experiments designed to analyze the concentration dependence of ACh-induced endothelial Ca^2+^ signaling, viable arteries were stimulated with various concentrations of externally applied ACh. In other experiments (described in the text), viable arteries were treated with pharmacologic inhibitors and incubated for 20 min, unless otherwise indicated. In these experiments, incubation was performed after control responses had been obtained. All responses were studied in paired experiments, and are expressed relative to control (maximal response for concentration response experiments).

### *En face* endothelial Ca^2+^ signaling and photolysis of caged inositol trisphosphate

Some experiments were not possible using the GRIN endoscopic imaging system [*e.g.,* caged inositol trisphosphate (IP_3_)]. In these experiments, Ca^2+^ signaling was examined in cut-open *en face* endothelial preparations, in which arteries were surgically opened and pinned out on a Sylgard block. Arteries were loaded with Ca^2+^ indicator loading solution or, for caged IP3 experiments, with Ca^2+^ indicator loading solution containing a membrane permeant caged IP_3_ [caged IP_3_ 4,5-dimethoxy-2-nitrobenzyl (DMNB); 10 µM] for 30 min at 37°C. Sylgard blocks were then placed face down on a 0 grade thickness microscope coverslips and the endothelium imaged using a wide-field epifluorescence microscope (TE2000U; Nikon). Steel pins (200 µm) were used as spacers to ensure the endothelium did not contact the coverslip. The Ca^2+^ indicator was excited with 488 nm wide-field epifluorescence illumination provided by a monochomator (Photon Technology International/Horiba UK, Ltd., Stanmore, United Kingdom) and fluorescence emission was collected by the objective lens (×40, numerical aperture 1.3) and transmitted to a cooled, back-illuminated electron-multiplying charge-coupled device camera (Cascade 512B; Photometrics, Tucson, AZ, USA) (28). Photolysis of caged IP_3_ was achieved as previously described (29–32), using a frequency tripled neodymium:yttrium aluminium garnet (Nd:Yag; wavelength 355 nm) laser attached directly to the microscope (Rapp Optoelektronic, Germany). The position of the photolysis site (∼2 µm diameter) was computer controlled (Rapp Optoelektronic, Hamburg, Germany). Images were recorded at 10 Hz. Identical UV flashes in the absence of caged IP_3_ evoked no response.

After loading and mounting, endothelial viability was confirmed by stimulating endothelial Ca^2+^ signaling was induced by application of 1 µM ACh. In experiments designed to analyze the concentration dependence of ACh-induced endothelial Ca^2+^ signaling in *en face* preparations, arteries were stimulated with various concentrations of ACh. In other experiments, silicone blocks were removed from the imaging chamber and arteries were preincubated with 500 µM Gap27 for 60 min at 37°C. Subsequently, arteries were placed back on the microscope and Ca^2+^ responses were recorded in the same field of cells. Responses were studied in paired experiments and are expressed relative to control (maximal response for concentration response experiments).

### *En face* endothelial immunocytochemistry

Surgically opened arteries pinned to Sylgard blocks were fixed in 10% neutral buffered formalin for 15 min at room temperature. Arteries were then washed 3 times in glycine solution, washed with PBS, permeabilized with Triton-X 100 (0.2% in PBS) for 15 min, washed again 3 times in PBS, and incubated with PBS containing 2% bovine serum albumin for 15 min, and then washed 3 times with PBS containing 0.05% Tween. Arteries were then incubated with a rabbit anti-muscarinic acetylcholine receptor (AChR) antibody (AB87199, 1:100; Abcam, Cambridge, United Kingdom) in PBS containing 2% bovine serum albumin for 1 h. After extensive washing in PBS containing 0.05% Tween, arteries were incubated with an Alexa Fluor 555-conjugated donkey anti-rabbit antibody (Invitrogen, Carlsbad, CA, USA) in PBS containing 2% bovine serum albumin for 1 h and then washed extensively in PBS containing 0.05% Tween. All processing steps and incubations were performed at room temperature. Arteries were then incubated in PBS containing 0.5 mg/ml of DAPI before being placed visualized on the same inverted microscope described above. Negative controls were performed in the absence of primary antibody.

In experiments where Ca^2+^ imaging and AChM_3_ receptor antibody staining were visualized in the same artery, endothelial Ca^2+^ signals were first evoked by bath application of ACh (30 nM; ∼EC_50_) and imaged as described above. Arteries were then removed from the microscope and stained for AChM3 receptor distribution and nuclear distribution. Arterial segments were then placed back on the microscope stage for visualization of the same region of endothelium in which Ca^2+^ signals were recorded. Images of Ca^2+^ activity and AChM_3_ receptor distribution were aligned using images of nuclear staining (DAPI). AChM_3_ receptor distribution was quantified, in endothelial regions that either responded or did not respond to 30 nM ACh, by summing background corrected AChM_3_ receptor staining intensity in the regions and dividing by the area of the region. For comparison across experiments, regional measurements were normalized against total staining in the total area of each of the images.

### Endothelial Ca^2+^ signal analysis

Endothelial Ca^2+^ imaging data were processed using a largely automated data processing procedure. First, regions of interest (ROIs) corresponding to individual endothelial cells were generated using a custom, semiautomated image processing segmentation procedure (Supplemental Fig. 1). In paired experiments, ROIs from control experiments were applied to response images. In concentration response experiments, the cell masks generated from maximal ACh concentrations were applied to all data sets. ROI alignment was manually checked for all datasets, and, in the event of movement between data sets were aligned using an automated alignment plug-in in ImageJ (National Institutes of Health, Bethesda, MD, USA) (33). Raw image stacks and ROIs were then imported into a custom Ca^2+^ signal analysis program implemented in the Python language. From each cellular ROI, individual fluorescence signals were extracted and stored as comma separated value (.csv) files. Fluorescence signals were then expressed as baseline corrected values (*F*/*F*_0_), calculated by dividing the raw signals by the average value of a 50-frame period preceding ACh-evoked Ca^2+^ activity. For an illustration of total Ca^2+^ activity, individual *F*/*F*_0_ traces were aligned with respect to their peak rate of change. The program automatically calculated baseline values of *F*/*F*_0_, peak amplitudes and the time of peak rate of change for each signal and stored them as .csv files. These files were then imported into Origin 9.1 (Silverdale Scientific, Ltd., Aylesbury, United Kingdom) for calculation of peak changes in fluorescence intensity (Δ*F*/*F*_0_), and for plotting using custom analysis scripts. An increase in cytoplasmic Ca^2+^ concentration ([Ca^2+^]_c_) was inferred when the peak *F*/*F*_0_ rose by more than 3 sd of baseline noise. As the choice of threshold affects the balance between detection rate and false positives, and depends on cellular loading and illumination/detection efficiency, baseline noise was calculated for individual cells over a 25 frame period preceding the onset of the rise in the *F*/*F*_0_ ratio. Curves were fitted to normalized concentration response data using GraphPad Prism 6.0 (GraphPad Software, La Jolla, CA, USA). The maxima and minima of the curves were constrained to unity and 0, respectively. Calculated curve-fit parameters (EC_50_) are presented with 95% confidence intervals.

### Solutions and drugs

The Ca^2+^ indicator loading solution consisted of the cell permeant acetoxymethyl ester form of Oregon Green 488 1,2-*bis*-(2-aminophenoxy)ethane-*N*,*N*,*N*′,*N*′-tetraacetate (OGB-1/AM; 20 μM), with a final concentration of 0.04% Pluronic F-127 and of 0.96% DMSO, in physiological salt solution. Drugs were all obtained from Sigma-Aldrich (St. Louis, MO, USA) except for the following: OGB-1/AM and Pluronic F-127 were obtained from Invitrogen, caged IP_3_ DMNB from SiChem (Berlin, Germany). The physiologic salt solution consisted of (mM): NaCl (145), KCl (4.7), 3-(*N*-morpholino)propane-sulphonic acid (2.0), NaH_2_PO_4_ (1.2) glucose (5.0), EDTA (0.02), MgCl_2_ (1.17), CaCl_2_ (2.0), pH adjusted to 7.4 with NaOH. Glycine solution consisted of MilliQ H_2_O containing 100 mM glycine (pH adjusted to 7.4 with NaOH). The PBS consisted of (mM): sodium chloride (137.0), potassium chloride (3.0) and disodium hydrogen phosphate (8.0), and potassium dihydrogen phosphate (1.5).

### Statistics

Summarized data are expressed as means ± sem. One-way nested ANOVA (with Tukey’s *post hoc* test as appropriate) was used for comparisons between groups. Biologic replicate (animal) was treated as a random effect. A value of *P* < 0.05 was considered significant.

## RESULTS

### Spontaneous endothelial Ca^2+^ responses

In arteries pressurized to 60 mmHg, spontaneously occurring, cellwide, transient increases in Ca^2+^ occurred infrequently (0.97% of cells from 154 separate imaging experiments). These spontaneous Ca^2+^ transients remained as solitary signaling events confined to particular cells and did not propagate even though substantial in magnitude (Fig. 1*F*, *G*).

### ACh responses

**Figure 2.**
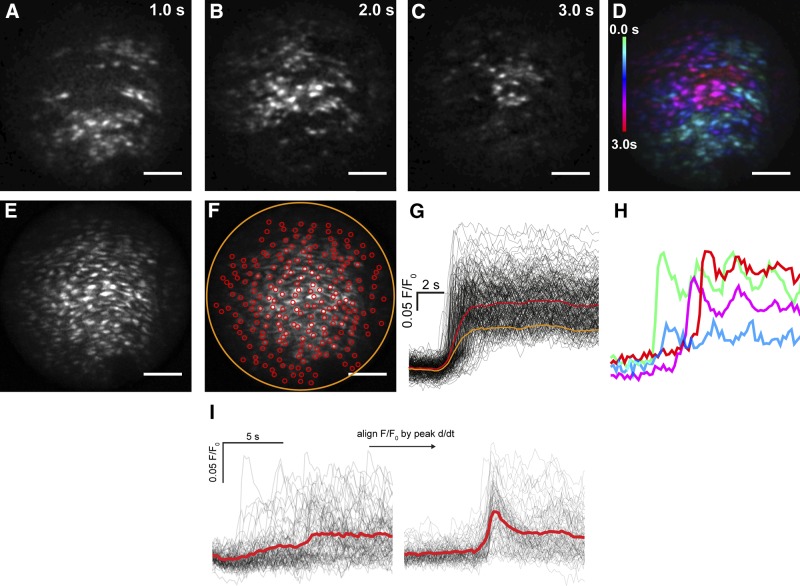
ACh-evoked Ca^2+^ signals in endothelial cells of an intact and pressurized rat carotid artery. In response to ACh, a Ca^2+^ increase initiated in one part of the endothelium and progressed from there. *A–C*) Images at 1-s intervals show active wave fronts of Ca^2+^ release as the wave progressed across the endothelium. The wave fronts were determined by sequential subtraction of image frames. *D*) A color-coded representation of the time of activation of the entire population of cells (blue early, red late). The time color-code is at left of image. *E*, *F*) Grayscale representation of all cells exhibiting Ca^2+^ activity (*E*) with individual manually placed circular ROIs encompassing single cells (red circles) and around the entire field (orange circle) (*F*). Scale bars, 100 μm. *G*) Cellular (black) and averaged (orange/red) Ca^2+^ signals from the ROIs. *H*) Selected Ca^2+^ signals from (*G*) mapped to color-code shown in *D*. *I*) Plotting ACh-evoked Ca^2+^ signals (left) from ∼200 cells illustrates the temporal heterogeneity of Ca^2+^ responses. Global mean data (red line) represents the data poorly. Ca^2+^ signals were differentiated and then aligned in time (right) with respect to the peak of the derivative Ca^2+^ signal, to synchronize the Ca^2+^ signals in each cell and illustrate total Ca^2+^ activity (thick red line).

Activation of the endothelium by extraluminal ACh [100 μM applied to the chamber, 100 nM estimated at the vessel lumen (see Supplemental Fig. 2); 60 mmHg Supplemental Movie 1)] evoked rises in [Ca^2+^]_c_ in the majority of cells in the field (**Fig. 2*A*–*D***). Within cells, repeating Ca^2+^ oscillations (uniform rises throughout the cell) and propagating Ca^2+^ waves (which moved at a velocity of 43 ± 3 µm/s; 60 cells from 6 arteries; not shown) occurred in various cells (Supplemental Movie 1). The Ca^2+^ rises evoked by ACh originated from an IP_3_-sensitive Ca^2+^ store. The Ca^2+^ increase persisted in a Ca^2+^-free bathing solution but was blocked by the sarco-endoplasmic reticulum Ca^2+^-ATPase inhibitor cyclopiazonic acid and the IP_3_ receptor blocker 2-aminoethoxydiphenyl borate (25). Caffeine failed to evoke a Ca^2+^ increase and ryanodine did not alter the ACh-evoked Ca^2+^ rise, suggesting ryanodine receptors play a minor role in Ca^2+^ signaling (25).

### Concentration-dependent ACh responses

To explore the organization of activity in the endothelium, we examined the concentration dependence of the endothelial response to ACh. The Ca^2+^ rise from each cell differed significantly in time of occurrence (latency; Fig. 2*A–H*) and duration giving rise to a substantial temporal spread of [Ca^2+^]_c_ elevations among cells (Fig. 2*D*, *G*, *H*) and complicating analysis. The large temporal spread rendered average measurements from the entire field (Fig. 2*F*, *G*, yellow lines) unrepresentative of the data and shows a monophasic elevation in [Ca^2+^]_c_. Responses from each cell were therefore examined by manually placing regions of interest on each cell (Fig. 2*F*, red circles). Although the overall rise (Fig. 2*G*, red line) was again monophasic, the amplitude was now more representative of the cellular responses. Therefore, single-cell responses were used in further analysis.

Identifying each cell manually in large-scale data sets presented a bottleneck in signal measurement, so a largely automated image-processing procedure was used to extract individual Ca^2+^ signals (Supplemental Fig. 1). The approach calculates the outline of each cell by using the activity of the cells themselves to facilitate identification (Supplemental Fig. 1). Each region of measurement was verified in each experiment. Using this approach, each cell’s Ca^2+^ response was rapidly extracted. However, the temporal spread of responses was particularly problematic at low ACh concentrations (Fig. 2*I*) where average responses appeared modest when compared with the responses of individual cells (Fig. 2*I*, left panel, red line). To overcome temporal spread, the [Ca^2+^]_c_ changes in each cell were aligned in time with respect to their peak rate-of-change (*i.e.,* first activation response) in a custom Python program. The aligned data illustrated the total Ca^2+^ response with greater clarity (Fig. 2*I*, right panel, red line).

However, when the data were temporally aligned, an unexpectedly large spread of amplitude of Ca^2+^ among cells, ranging from no change to a maximum response, was apparent (Fig. 2*I*, right panel) (7, 9, 34, 35). Therefore, rather than averaging responses of all cells at each concentration, the Ca^2+^ rise of each cell at each concentration was examined separately.

**Figure 3.**
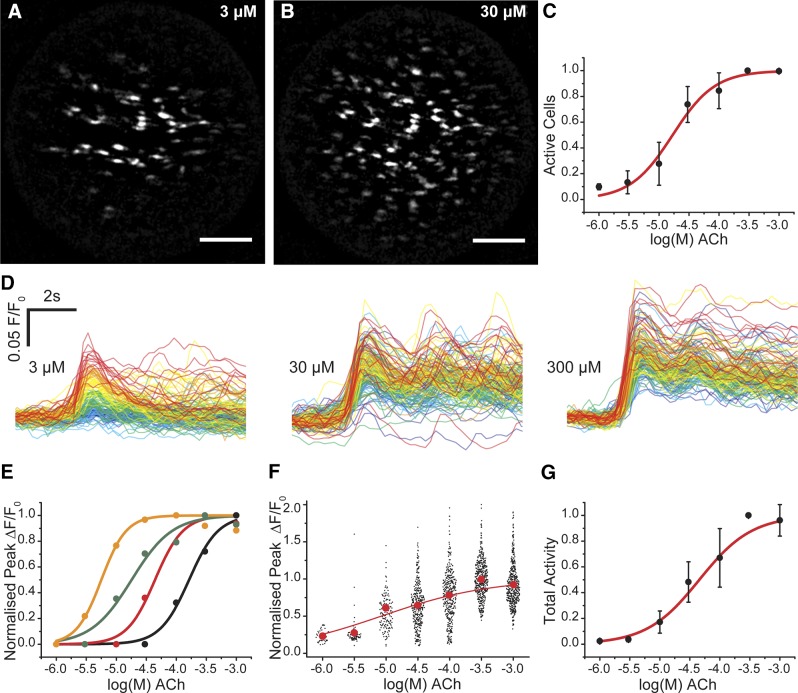
Graded concentration responses to ACh. *A–C*) As the concentration of ACh increased, the number of cells activated increased. *A*, *B*) Maximum intensity projections show the total number of cells activated at 3µM (*A*) and 30 µM (*B*) ACh. As the concentration of ACh increases, more cells are activated. Scale bars, 100 μm. *C*) Summarized data showing number of active cells (EC_50_ = 18.9 μM; 95% confidence interval, 7.25–49.4 μM; *n* = 3). *D*) The amplitude of response in each cell also increased with concentration of ACh. The responses to 3 illustrative concentrations from the full concentration–response relationship are shown. The responses in each cell have been time-aligned and color-coded based on ACh sensitivity of the cells at the lowest ACh concentration: red, most sensitive; blue, least sensitive. As the concentration of ACh increases, the amplitude of the responses increases. There is overlap in the response between 30 and 300 μM because of the position in the concentration response relationship. *E*) Representative concentration responses from 4 cells in 1 experiment that show a range of sensitivities to increasing ACh concentration. *F*) Scatter plot of the overall responses from 445 cells from 3 arteries. The red dots plot the mean response at each concentration. The overall relationship appears flat because all responses at each concentration from separate arteries are shown. *G*) Total endothelial responses (EC_50_ = 42.7 μM; 95% confidence interval, 20.2–90.1 μM; *n* = 3) derived from the product of the number of active cells (*C*) and mean response (*F*) at each concentration.

Taking this approach, several unique features of the endothelium’s response to increasing concentration of ACh were now apparent (**Fig. 3**). First, at low concentrations only a small number of cells activated (sensitive cells; Fig. 3*A* and Supplemental Movie 2). As the agonist concentration increased, additional cells were recruited in a concentration-dependent manner (Fig. 3*A–C* and Supplemental Movie 2). Second, after recruitment, the amplitude of [Ca^2+^]_c_ response within each cell also increased in a concentration-dependent manner (*i.e.,* each cell responded with a typical concentration–response relationship) (Fig. 3*D–F*). However, individual cells operated over various concentration ranges (Fig. 3*D*, *E*). The overall response of the endothelium was derived from the combined, separate, concentration sensitivity of each cell of the population (Fig. 3*G*). Each cell’s responsewas constrained to <10^2^ concentration (Fig. 3*E*), though the overall response of the endothelium was spread over 3 orders of magnitude of concentration (Fig. 3*G*). These results suggest that endothelial cells are primed with a limited range of sensitivity that varies significantly among cells. The combined activity determines the amplitude of Ca^2+^ response to provide a detection system with both high sensitivity and wide dynamic range.

**Figure 4.**
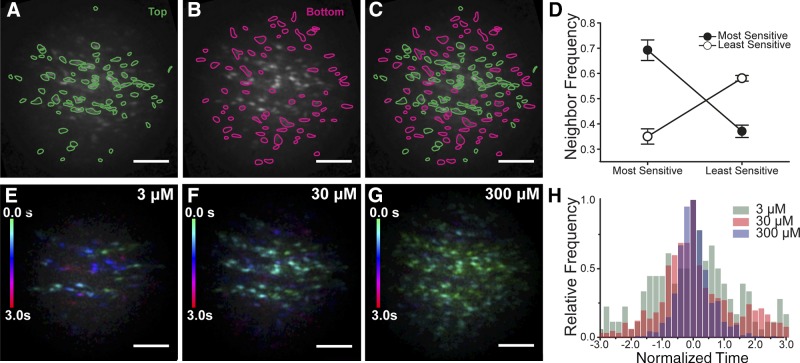
Endothelial cells cluster based on sensitivity and activate in spatially discrete ensembles. *A–D*) Cells of comparable sensitivity cluster. *A*) Plot of the grouping of cells with the highest sensitivity to ACh (top 50%). *B*) Plot of the cell grouping with the least sensitivity to ACh (bottom 50%). *C*) Composite of plots *A* and *B*. *D*) Plots of neighbor frequency for the high- and low-sensitivity cells normalized to the mean number of neighbors of all cells. Cells of a given sensitivity type (high or low) have significantly more neighbors of the same type. The most sensitive cells (top 50%) have more most-sensitive neighbors and fewer least-sensitive (bottom 50%) neighbors. The least-sensitive cells (bottom 50%) have more least-sensitive neighbors and fewer most-sensitive (top 50%) neighbors. *E–G*) Maximum intensity projection Ca^2+^ wave fronts showing the number of active cells at each concentration. At each concentration, all cells that respond are shown. Cell activity has been color-coded based on the time each cell is activated (green, early; red, late; color scale left side of each frame). The temporal Ca^2+^ pattern to 3 concentrations of ACh (3 µM, *E*; 30 µM, *F*; 300 µM, *G*) from the same cells shown in the upper panels (*A–C*). In each panel (*E–G*), all cells that respond to ACh are shown. At the lowest concentration (3 µM) a small number of cells initially respond (green cells). These cells then recruit some additional cells (purple and red cells). As the concentration of ACh increases (30 µM, *F*) more cells are activated initially (green), and these recruit additional cells (blue). At the highest concentration, almost all cells respond almost immediately (green). The decrease in time for activation of the field reveals the increased recruitment of lower sensitivity cells with higher ACh concentrations. Scale bars, 100 µm. *H*) Normalized frequency distribution illustrating the decreased temporal spread of cellular activation with increasing concentrations of ACh. Three increasing concentrations of ACh (3, 30, and 300 μM; *n* = 6) are shown. The data are a normalized frequency distribution showing the time to peak response for each cell in the endothelium.

Examination of the distribution of cells with low and high sensitivity shows an apparent clustering of cells with comparable sensitivities (**Fig. 4*A–C***). Although the pattern is not completely homogenous, where there is a high-sensitivity cell, there is a high probability that a neighboring cell is a high-sensitivity cell (Fig 4*D*). Where there is a low-sensitivity cell, there is a high probability of a neighboring cell being a low-sensitivity cell. For this analysis, from the Ca^2+^ signals, cells were split into the most sensitive 50% (red) and least sensitive 50% (green) (Fig. 4*A–C*). From the image masks, the center of each cell was identified (barycenter of polygon image masks), and the average distance from each cell mask center to its nearest neighboring center was determined (∼16 pixels). A search was then performed within twice this radius (32 pixels), to determine how many cells fall within that distance. The search was limited to exclude the outer 32 pixels of our circular image to prevent bias from edge effects (*e.g.,* measurement from outside cells). For each of the high-sensitivity and low-sensitivity cells, the number of high-sensitivity and low-sensitivity neighboring cells within 32 pixels were measured separately (Fig. 4). In both cases, cells of a given sensitivity type (high or low) have significantly more neighbors of the same type. This data suggest that the distribution of sensitivity to ACh is spatially structured by like-sensitivity cells being clustered. The clustering creates a multicellular equivalent of the receptor field (36) that occurs in other sensory systems from which detected signals are relayed. Indeed the multicellular clustering in the endothelium appeared also to be coupled to function and to transmit Ca^2+^ signals to neighboring cells as waves (Fig. 2*D*). The temporal response to ACh activation and spread of the Ca^2+^ signal were therefore next examined.

At low agonist concentrations, small spatial groupings of cells (macrodomains), which were often arranged in strips following the length of the artery [also see Huang *et. al*. (7)], activated (Fig. 4*D* and Supplemental Movie 2). After the initiating ensemble activated, Ca^2+^ rises expanded to neighboring cells (Fig. 4*E*). In the images (Fig. 4*E–G*), all cells that responded are shown. Interestingly, as the concentration of ACh increased, more cells within the initiating ensemble responded simultaneously (Fig. 4*F*, *G*). These additional cells were the same cells previously activated by secondary wave propagation at lower concentrations of ACh. At higher ACh concentration, the [Ca^2+^]_c_ rise also spread further to neighboring cells as Ca^2+^ waves, the temporal spread of the response decreased (Fig. 4), and the remaining majority of cells in the field-of-view were recruited at high agonist concentration. These waves may offer a means for communication over distances.

**Figure 5.**
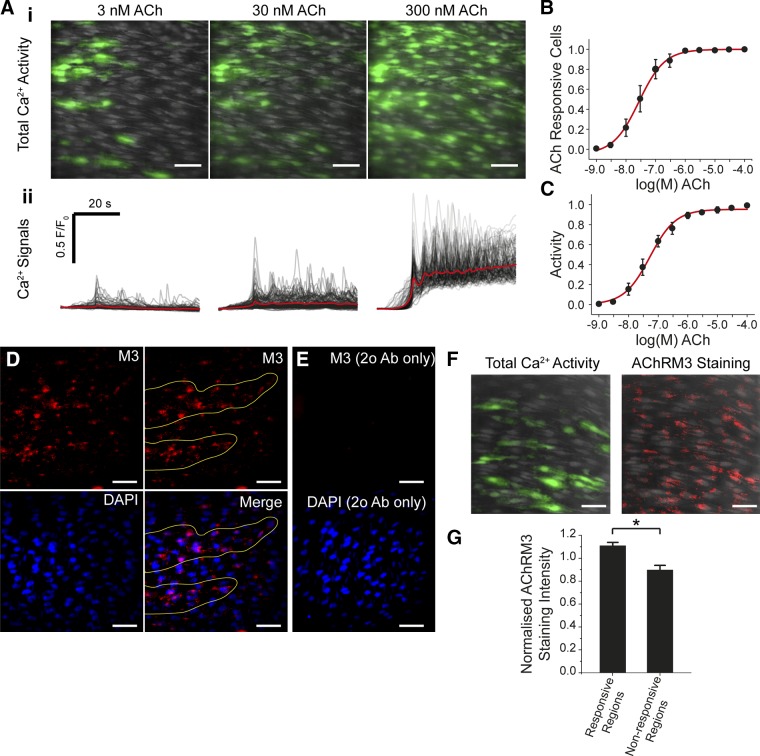
ACh-evoked Ca^2+^ responses and distribution of muscarinic receptors. (*A*) ACh-evoked Ca^2+^ signaling in the *en face* endothelial preparation. *A*) *i*) Images illustrating a field of endothelial cells and Ca^2+^ rises (green) in response to ACh [3 nM (left), 30 nM (middle), and 300 nM (right)]. Ca^2+^ rises occurred in discrete clusters of endothelial cells at lower ACh concentrations and in the majority of the field at the higher concentration. The cells shown are average images (gray) with Ca^2+^ activity overlaid (green). *ii*) Line trace of the Ca^2+^ changes from the same fields and ACh concentrations shown in *i*. *B*) Summarized data showing the total number of ACh responsive cells at each ACh concentration (EC_50_ = 27.3 nM; 95% confidence interval, 18.0–41.2 nM; *n* = 4). *C*) Total endothelial responses (activity) derived from the product of the number of active cells and mean response at each concentration) (EC_50_ = 52.5 nM; 95% confidence interval, 41.0–97.1 nM; *n* = 4). *D*) Immunohistochemical localization of endothelial M3 muscarinic AChR M3 in the endothelium of *en face* arterial preparations. Representative image (top left) illustrating that AChR M3 distribution was not uniform across the endothelium but more densely clustered to discrete regions (*D*, top right; yellow lines). In the same preparation, nuclei of endothelial cells were labeled with DAPI (*D*, bottom left). An overlay (*D*, bottom right) of endothelial nuclei (blue) with AChRM3 (red) staining shows the clustered localization of AChRM3 to particular regions of endothelium (bottom right; yellow lines). *E*) Negative control obtained by omitting the anti-AChRM3 (E, top panel). DAPI loading (*E*, bottom panel) shows cell nuclei positions. *F*) AChRM3s have increased occurrence in sensitive cells activated by ACh. Left panel, total endothelial Ca^2+^ activity (green; evoked by 30 nM ACh, left panel) overlaid on cells (gray) in an *en face* preparation. Right panel, immunohistochemical localization of endothelial AChRM3 (red) in the same field of endothelium (right panel) previously activated with ACh. Nuclei are shown in blue (DAPI staining). *G*) Summary data showing that AChRM3 are more densely localized in sensitive regions of endothelium (*i.e.*, green in *F*) compared with those regions that are less sensitive to ACh (*n* = 3). *P < 0.05. Scale bars, 50 μm.

The variation in sensitivity to ACh in different regions of the endothelium may be explained by variations in the density of the ACh receptor (AChR) population. Regions most sensitive to ACh had a higher density of the muscarinic AChR M3 (**Fig. 5**). In initial experiments, similarities in the pattern of sensitivity to ACh and the distribution of AChRM3 was noted (Fig. 5). Subsequently, the pattern of endothelial Ca^2+^ signaling evoked by ACh and the distribution of AChRM3 receptors was examined in the same artery. Those regions most sensitive to a half-maximal effective concentration of ACh (30 nM) had a significantly (*P* < 0.05) increased number of AChM3 receptors (Fig. 5).

### Chaotic signals: Ca^2+^ wave transmission or sequential cell activation by ACh?

**Figure 6.**
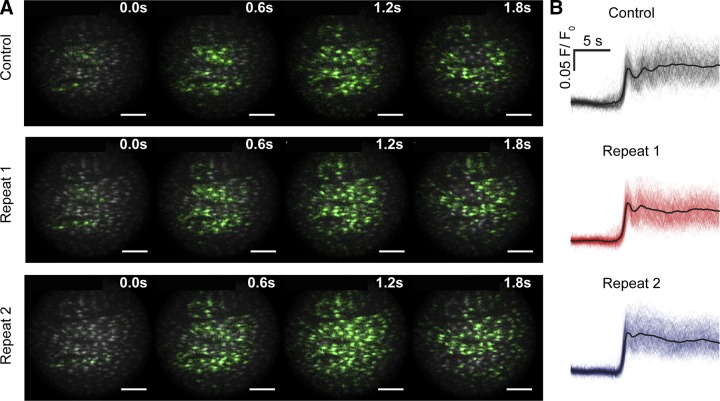
IP_3_-mediated endothelial Ca^2+^ response evokes complex but repeatable spatiotemporally signals. *A*) Time series fluorescence images of active Ca^2+^ wave fronts showing the progression of ACh (100 µM; bath applied) Ca^2+^ waves across the endothelium. In 3 separate applications of ACh in the same artery, approximately reproducible Ca^2+^ patterns of signal progression occurred. Images are composed of instantaneous Ca^2+^ activity (green) overlaid on standard deviation images (grayscale) indicative of total Ca^2+^ activity. Scale bars, 100 µm. *B*) Automatically extracted and temporally aligned Ca^2+^ signals from data shown in (*A*).

Ca^2+^ waves arising from the initiator ensemble appeared to preferentially travel along the longitudinal axis of the vessel when initially activated by ACh. However, as the paths of Ca^2+^ waves from different initiating sites crossed, annihilation occurred on collision and the initial large-scale Ca^2+^ waves decoupled into complex spatiotemporal patterns of Ca^2+^ signaling (Supplemental Movie 1). Although Ca^2+^ signaling appeared disordered, the pattern of initial wave progression was consistent on repeated activations (**Fig. 6** and Supplemental Movie 3). When the artery was activated consecutively, the average amplitude of the Ca^2+^ signal in all cells was 0.99 ± 0.04% (*n* = 7) of control. The reproducible nature of the disordered wave appears to suggest a signal may be encoded in the pattern.

**Figure 7.**
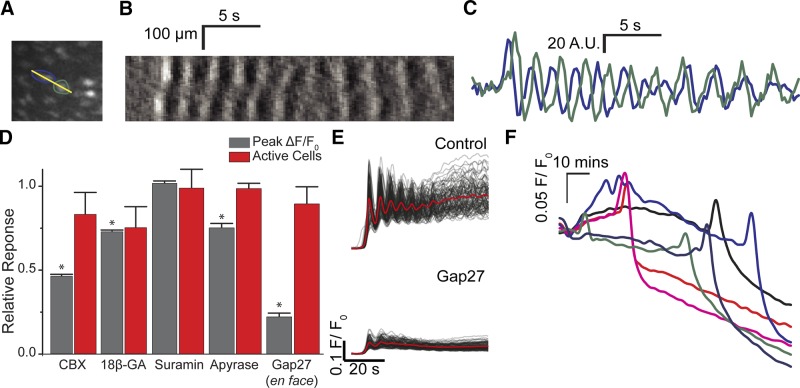
Dynamics of Ca^2+^ wave propagation. Ca^2+^ waves appear to progress both within and between cells. *A*) Ca^2+^ signaling activity between endothelial cells. *B*) A kymograph (across yellow line in *A*) shows apparent bidirectional transmission of Ca^2+^ waves (rises in [Ca^2+^]_c_ are shown as lighter shades and declines are shown as darker shades) across 2 endothelial cells. The sequence of activation is not constant revealing the changing direction of the wave. *C*) Plots of Ca^2+^ signal from the same 2 cells (*A*, *B*) show the changes in timing of the 2 signals. The time of Ca^2+^ rise changes. *D*) The effects of Gap junction and ATP receptor blockers on the ACh-evoked endothelial response. Normalized summary data showing the average amplitude of the peak ACh-evoked (100 μM) response (gray) and the number of cells activated (red). The average ACh-evoked endothelial Ca^2+^ response, but not the number of activated cells was significantly reduced by CBX (100 μM, *n* = 3) and 18βGA (100 μM, *n* = 3). ACh-evoked responses persisted in the presence of suramin (100 μM, *n* = 3) and apyrase (4 U/ml). Gap27 (500 µM), a peptide-based connexin 43 mimetic, reduced the amplitude of the Ca^2+^ responses in each cell activated but not the number of activated cells. Data presented as means ± sem and normalized to control (no treatment, 1; not shown), *P < 0.05. *E*) Baseline-corrected ACh-evoked endothelial Ca^2+^ signals (*F*/*F*_0_) from ∼150 cells in the absence (top) and presence (bottom) of Gap27. *F*) Baseline-corrected (unaligned) endothelial Ca^2+^ signals (*F*/*F*_0_) from 6 cells in an unstimulated artery incubated with 18βGA (100 μM). Recordings are from a time-lapse experiment in which images were acquired at 10-s intervals, and start approximately 60 min after introduction of 18βGA. Signals in both panels have been temporally smoothed with a 10-point running average. The blockers, by themselves, cause a significant Ca^2+^ rise after prolonged incubation.

After the initial Ca^2+^ waves decoupled into multiple spatially restricted events, the Ca^2+^ changes did not subsequently entrain and synchronize, to become a uniform oscillation throughout the endothelium, or co-ordinate to move in a particular direction. However, waves still appeared to progress within cells, and close inspection revealed small groupings of cells that appeared to remain linked by the apparent transmission of the oscillatory Ca^2+^ waves between cells (**Fig. 7*A–C***). However, the direction of wave travel between the apparently linked cells was not fixed and could reverse rapidly (Fig. 7*A****–****C*). These observations raised the question of whether cells were coupled and acted as conduit for Ca^2+^ signal progression along the endothelium or if cells were completely uncoupled and the Ca^2+^ rises in each cell were independent but temporally coincident to create the impression of wave progression. Coincidental sequential activation could, for example, arise from the time required for the concentration of ACh to change after addition and the various sensitivities of cells to ACh.

To determine if waves progressed through cells rather than the Ca^2+^ rises being a coincidental but separate activation of neighboring cells, the Gap junction blockers carbenoxolone (CBX; 100 µM) and 18β-glycyrrhetinic acid (18βGA, 100 µM) were used in separate experiments. Each blocker substantially reduced the response to ACh (Fig. 7*D*). However, the Gap junction blockers may have acted nonspecifically to inhibit Ca^2+^ waves. Prolonged exposure to either 18β-glycyrrhetinic acid (100 µM) or CBX (100 µM) blocked all response to ACh after 40 min, and the drug itself caused a slow increase in [Ca^2+^]_c_ that was followed by a decline to below resting values after approximately 2 h (Fig. 7*E*). These results suggest that the blockers may have a wide spectrum of activity beyond effects on Gap junctions. Pannexins (Panx1) are inhibited by CBX and 18βGA acid (37) at concentrations similar to those used in the present study. Panx1 may release signaling molecules such as ATP from the cytoplasm to the extracellular space (38). However, in other experiments, ACh-evoked Ca^2+^ waves were not inhibited by suramin (100 µM) or apyrase (4 U/ml), which suggests that Panx1 and regenerative ATP release are unlikely contributors to wave progression (Fig. 7*D*). In other experiments the effects of the inhibitory connexin-mimetic peptide, Gap 27 (500 µM), was examined. In an *en face* preparation, Gap 27 (500 µM; Fig. 7*D*, *E*) reduced the amplitude of ACh (1 µM) evoked Ca^2+^ signals in each activated cell. However, the number of cells activated by ACh was unaltered in the presence of Gap27 (Fig. 7*D*, *E*).

**Figure 8.**
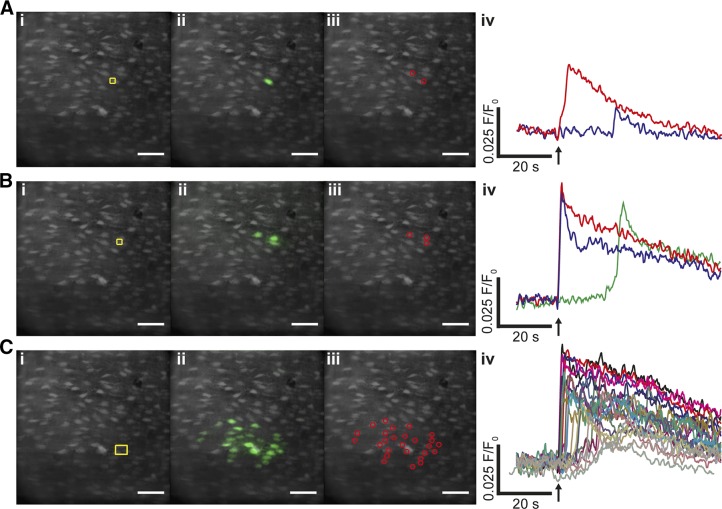
Local photolysis of caged IP_3_ triggered [Ca^2+^]_c_ increases, which propagate to neighboring cells. *A*) In a field of endothelial cells, locally photolyzed caged IP_3_ in a single endothelial cell (*i*, yellow box) increased [Ca^2+^]_c_ in the activated cell (*ii*, green; *iv*, red) and a smaller response in an adjacent cell (*iv*, blue). The time course of the [Ca^2+^]_c_ changes, from the cells indicated by the regions of interest (*iii*), are shown in the line trace (*iv*). Immediately after photolysis (*iv*, ↑), a Ca^2+^ rise occurred in the activated cell, and a subsequent rise occurred in an adjacent cell some 20 s later. The Ca^2+^ changes in *ii* show all Ca^2+^ activity (*i.e.*, each cell that responds is shown irrespective of time of response). *B*) IP_3_ photoreleased simultaneously in 2 adjacent cells (*i*, yellow box) evoked a Ca^2+^ rise in each of the activated cells (*ii*, green; *iv*, red and blue lines) and Ca^2+^ rise in a neighboring cell (green) some 20 s later. The time course of the [Ca^2+^]_c_ changes, from the cells indicated by the regions of interest (*iii*), are shown in the line trace (*iv*). *C*) Photolyzed caged IP_3_ in 4 cells simultaneously (*i*, yellow box) evoked a local Ca^2+^ increase, which rapidly propagated to a substantial number (∼18) of neighboring cells (*ii*–*iv*). The Ca^2+^ changes extended several cell lengths from the photolysis site (*i*, *ii*). The time course of the [Ca^2+^]_c_ changes, from the cells indicated by the regions of interest (*iii*), are shown in the line trace (*iv*). Scale bars, 50 µm.

Because the Gap junction blockers had limited use in determining if cells were coupled to progress waves among cells, another approach was taken to study cell–cell communication. Specific preselected cells were activated by locally photolyzing caged IP_3_ and the transmission of the Ca^2+^ signal from these cells was then examined (**Fig. 8**). Photolysis of caged IP_3_ in a single cell triggered a rapid rise in [Ca^2+^]_c_ in the activated cell (Fig. 8*A*). After a significant delay (∼8 s) a small rise in Ca^2+^ occurred in a neighboring cell (Fig. 8*A*). The amplitude and spread of wave propagation to neighboring cells was determined by how many cells were activated simultaneously. Photorelease of caged IP_3_ in 2 cells simultaneously (Fig. 8*B*) triggered an immediate rise in [Ca^2+^]_c_ in the activated cell and a [Ca^2+^]_c_ rise in a neighboring cell. That [Ca^2+^]_c_ rise was more substantial than occurred when only a single cell was activated (Fig. 8*A*). When 4 cells were activated simultaneously by photolysis of caged IP_3_ (Fig. 8*C*), a substantial [Ca^2+^]_c_ rise propagated rapidly to a substantial number (∼18) of neighboring cells (Fig. 8*C*) and extended several cells lengths from the photolysis site. These experiments demonstrate that Ca^2+^ rises triggered in a single cell propagate to neighboring cells, presumably *via* direct cell–cell communication. The extent of propagation is determined by the number of cells simultaneously activated and offers a coincidence detection system that increases signal fidelity. Similar results were seen on 3 other experiments. Although these results do show cell–cell communication, the mechanisms of wave transmission arising from release of caged IP_3_ may not necessarily be the same as that of ACh-evoked Ca^2+^ waves, and the route of transmission between cells is not clear from the present findings.

## DISCUSSION

**Figure 9.**
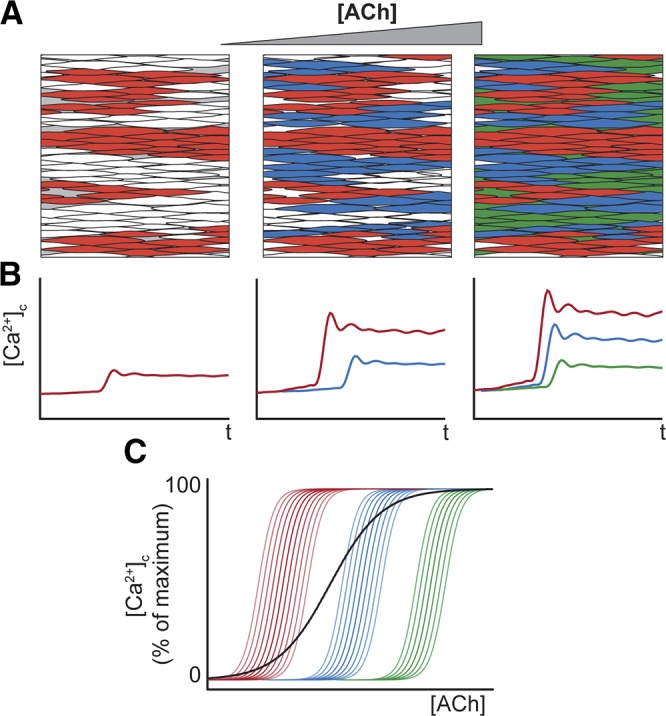
Multicellular signaling macrodomains (receptive field) provide high-sensitivity, wide dynamic-range agonist sensing in the vascular endothelium. Clusters of cells positioned close to each other have various sensitivities to ACh. As the ACh concentration increases (*A*), the most-sensitive cells activate first. (*B*, *C*). Less-sensitive cells (blue, green) activate at higher ACh concentrations. Over their sensitivity range, each cell responds with an ACh-concentration-dependent increase in [Ca^2+^]_c_ (*B*, *C*). The combined activity of each of the cells provides the overall response of the endothelium (*C*, black line) and a high-sensitivity response over a wide concentration range.

The environment in which the endothelium operates is a confusion of multiple agonists whose concentrations change almost continuously. Yet the endothelium sensitively discriminates very small changes in agonist concentration while being able to exclude random noise. The endothelium also provides responses that span a wide concentration range. How these normally incompatible detection features (insensitivity to noise, high sensitivity to signal, wide concentration sensitivity range) are reconciled and achieved is not well understood. Our results from large areas (∼200 cells) of endothelium in intact, pressurized arteries show the endothelium achieves high sensitivity, robustness to noise, and wide concentration range detection by integration of populationwide heterogeneous responses. Individual cells contribute only a small aspect of the overall features of detection. Each cell responded to chemical activation (ACh) with concentration-dependent increases in Ca^2+^ activity that spanned a single order of agonist-concentration magnitude. Intercellular variation in EC_50_ values (3 orders of magnitude) increased the bandwidth of concentration sensitivity and enabled collective endothelial responses to both low- and high-intensity stimuli. Cells with comparable sensitivity clustered in restricted spatial domains. This clustering acted as a noise rejection system by acting as a “coincidence detector.” Simultaneously activating several cells in the clusters triggered Ca^2+^ signals that transmitted to neighboring cells in a manner that scaled with stimulus intensity (**Fig. 9**). Thus, cell clustering and signal propagation provide an integrative mechanism for robust noise filtering and agonist sensing.

In other cell types, several proposals have been made to explain high sensitivity to low agonist concentration and maintained sensitivity and responsiveness at high agonist concentrations. Osteoclasts use several different purinergic receptor subtypes, each with various affinities, to provide responses to ATP over a concentration range from 1 nM to 1 mM (39). However, the endothelium largely relies on a single receptor muscarinic receptor subtype (AChRM3) yet retains a wide agonist sensitivity range. To provide sensitivity with a single receptor subtype, some cells may use a binary (discrete) type response in which the number of cells respond with quantal (all-or-none) responses and the number of active cells (40, 41) or the frequency of Ca^2+^ transients in the population (42) scale with concentration because of the agonist sensitivity set in each cell. This would be effective in compensating for variations in the background concentration so that cells remain sensitive to fluctuations in agonist concentrations around basal level while also being able to respond to increasing concentrations. However, because a single cell represents a single piece (bit) of information only, this type of binary logic may be unable to reproduce complex networked signaling typical of the endothelium systems because of the finite numbers of cells involved (43) (*e.g.,* dynamic signaling range and signal complexity will be limited) or only low resolution to changes in concentration may be available. Another possibility is that graded responses within each cell may scale with agonist concentration to provide the concentration-dependent response (44). In this case, each cell may provide several bits of information in signal processing and act as a type of analog signal processing unit. However, in this type of analog logic, if all cells responded equally, the endothelium may be more sensitive to noise, have difficulties discriminating concentrations just above background levels, and have a limited concentration range over which the cells may operate (*i.e.,* sensitivity will be limited). The endothelium combines elements of each proposal. Although each cell responds with an analog concentration-dependent response, the sensitivity difference in various cells overcomes the limited single-cell bandwidth to provide a detection capability at low and high agonist concentration. Differences in agonist-sensitivity in cells also generates a concentration dependence to the number of active cells and, as a result, provide a significant number of binary or digital (discrete) combinations that may contribute to the overall response.

The positioning of cells with comparable sensitivity close together provides a coincident detection facility and may reduce the likelihood of spurious signals being transmitted (Fig. 9). This clustering creates a spatial separation of sensitivity classes of sensing cells. Although signaling microdomains, in which proteins are concentrated to a specific region within the endothelial cell, are now well established (3, 5, 45), the present results suggest that multicellular signaling macrodomains also occur in which cells with similar activity are positioned together. These macrodomains create a sensory space in which a stimulus elicits a response and so resemble multicellular receptive fields (36) of other sensory systems. The endothelium’s receptive field relays information to neighboring cells and provides a means to sense specific stimuli in a crowded chemical environment in which extracellular activator concentrations fluctuate constantly around background levels. The various sensitivity levels are explained at least in part by variation in the number of muscarinic receptors expressed. How endothelial cells of comparable sensitivity cluster is unclear, but perhaps self-replication occurs during development or there is feedback control of function and receptor expression based on location.

Endothelial cells are coupled *via* Gap junctions. Small molecular weight components can diffuse among cells *via* Gap junctions (8, 46), albeit at very slow rates, which may permit electrical coupling (47) and propagation of Ca^2+^ signals (48, 49). Endothelial cells are also coupled with smooth muscle cells (5, 50, 51). However, interestingly, despite the apparent high level of connectivity, in the present study spontaneous Ca^2+^ release events remained confined to single cells even though the Ca^2+^ rises were substantial in magnitude. On the other hand, immediately after activation with ACh, Ca^2+^ rises of similar magnitude to spontaneous events were apparently transmitted as coordinated waves, which progressed through the endothelium (*i.e.,* cell–cell communication). The question arises, why are spontaneous events not transmitted when activated Ca^2+^ rises are not? One possibility is that the wave does not reflect cell–cell communication but rather sequential activation of cell with various ACh sensitivity. Indeed, after the initial wave on ACh application, the coordinated wavelike behavior is lost rapidly and the links between cells decoupled giving rise to asynchronous Ca^2+^ changes in various cells (Supplemental Movie 1). Asynchronous Ca^2+^ oscillations and Ca^2+^ waves have been previously seen in endothelial tubes and sheets (34). However, support for the existence of multicellular transmission of Ca^2+^ waves was found in experiments that used localized flash photolyzed caged IP_3_. Release of IP_3_ in specific preselected cells triggered a Ca^2+^ rise that was transmitted from the release site and among several cells. The mechanism of propagation may involve IP_3_ or Ca^2+^ or both.

The wide control the endothelium exerts over cardiovascular structure and function requires endothelial cells to sense stimuli effectively, to have a wide sensitivity range to activators, and to communicate efficiently both with itself and other cell types. The endothelium is exposed to hundreds of chemical modulators and detects autocrine and paracrine signals with exquisite sensitivity. These extracellular activators are transduced to intracellular (Ca^2+^) signals to regulate several vascular activities including smooth muscle contraction and proliferation. The sensing systems for extracellular activators are likely to vary through the vascular system (*e.g.,* arteries, vein) and between larger arteries and capillaries and with mechanical stimuli like pulsatile pressure. The results presented in intact arteries at a constant pressure suggest the endothelium detects extracellular activators by acting as a distributed sensing system that is organized into separate clusters of functionally coupled detector cells (macrodomains). The macrodomains act as linked relay elements of a communicating network, and excitation patterns are connected to the stimulus concentration. Although each endothelial cell is a detector, cells are set with a fixed and limited sensitivity to activators. The overall response is derived from the merged responses of many different cells with various agonist sensitivities to achieve a consensus on agonist concentration. Cells are able to detect small fluctuations in activator concentration around basal levels because cells of comparable sensitivity are clustered to provide a coincidence detection system. The extent of signal propagation depends on how many neighboring cells are activated simultaneously. After activation, propagation of signals provides a means to communicate sensed information across physical scales relevant to the artery *via* IP_3_ or Ca^2+^ waves. The propagating intercellular waves show complex progression patterns and appear chaotic initially because of the multiple directions of progression and annihilation when waves collide. Yet the pattern is repeatable on successive applications of ACh, which suggests an encoded signal. The complex interaction of bioactive molecules with spatial domains and cell–cell communication suggests that electrically nonexcitable endothelial cells function together as an artery-lining macroscopic network that enables scalability in highly sensitive responses.

## Supplementary Material

Supplemental Data

## Abbreviations

ACh: acetylcholine
AChR: acetylcholine receptor
[Ca^2+^]_c_: cytoplasmic Ca^2+^ concentration
CBX: carbenoxolone
GRIN: gradient index
IP_3_: inositol trisphosphate
OGB-1/AM: Oregon Green 488 1,2-*bis*(2-aminophenoxy)ethane-*N*,*N*,*N*′,*N*′-tetraacetate acetoxymethyl ester
ROI: region of interest
