# Supplementary material for: Clusters of specialized detector cells provide sensitive and high fidelity receptor signaling in the intact endothelium
Source: FASEB J. 2016 Feb 12;30(5):2000–13. doi: 10.1096/fj.201500090 (PMC4836367; doi:10.1096/fj.201500090)
Supplement: Supplemental Data [file supp_30_5_2000__index.html]

Clusters of specialized detector cells provide sensitive and high fidelity receptor signaling in the intact endothelium — Supplemental Data 

# Clusters of specialized detector cells provide sensitive and high fidelity receptor signaling in the intact endothelium

## Supplemental Data

- Supplemental Data
- Supplemental Data
- Supplemental Data
- Supplemental Data
