# Supplementary material for: Clusters of specialized detector cells provide sensitive and high fidelity receptor signaling in the intact endothelium
Source: FASEB J. 2016 Feb 12;30(5):2000–13. doi: 10.1096/fj.201500090 (PMC4836367; doi:10.1096/fj.201500090)
Supplement: Supplemental Data [file supp_fj.201500090_Supplemental_Figures1.pdf]

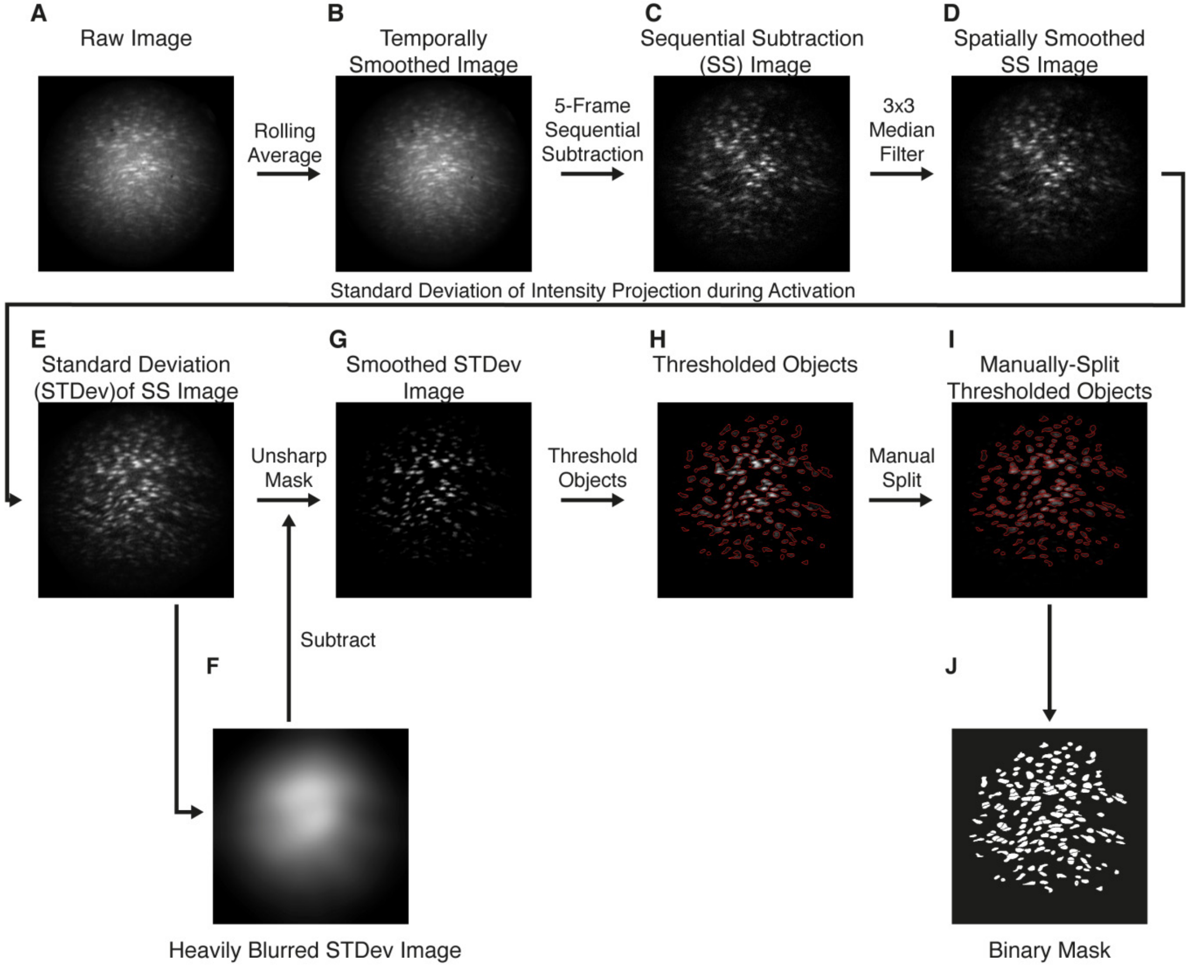

Supplementary figure 1. Largely automated segmentation of individual cells from endothelial Ca^{2+} imaging data. (A) Raw image of rat carotid artery endothelial cells, loaded with OGB-1/AM, obtained from an intact, pressurized artery. (B) Temporally smoothed image. (C) 5-Frame sequential subtraction of (B), showing endothelial Ca^{2+} wave activity. (D) Noise was reduced by applying a 3x3 pixel median filter to (C). (E) Projection of the standard deviation of intensity of (D) during activation. (G) Cleaned image of (E), obtained by subtracting a heavily blurred (25x25 mean filter, 10 passes) copy (F) of (E) from (E) itself, followed by a single pass of a 3x3 median filter. (H) Cell outlines obtained by threshold segmentation shows that some cells are not split correctly. (I) Individual cells were obtained by manually splitting objects in (H). (J) Binary image mask of individual cells

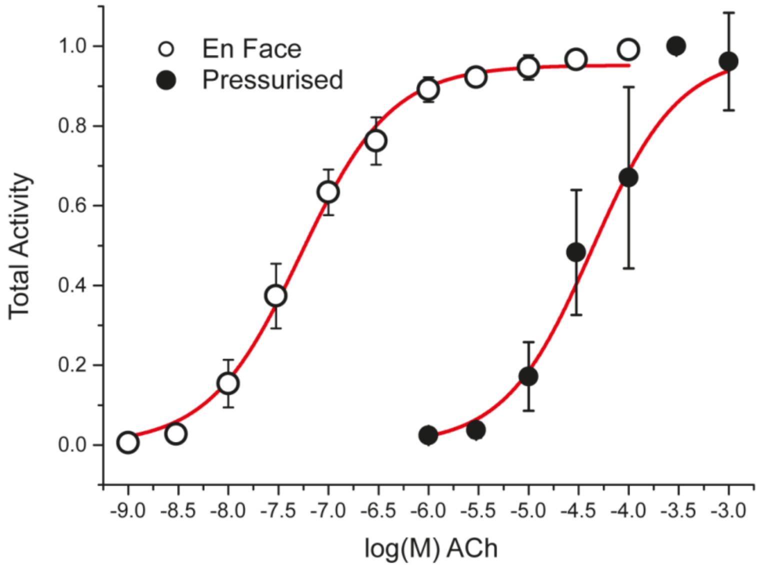

Supplementary figure 2. Endothelial Ca^{2+} concentration response to ACh in pressurized arteries and surgically-opened en face arteries. ACh applied to pressurized artery had to diffuse through the outside wall to reach the endothelium. On the other hand, there were no diffusional restrictions in the surgically-opened *en face* preparation. The endothelium was more sensitive to ACh in the *en face* artery than in the pressurized artery and the EC_{50} ~1000 fold less. Ca^{2+} changes are shown as total activity which is derived from the product of the fraction of cells active and normalized mean response at each concentration.
